# International diet quality index and revised diet quality index relationship with type 2 diabetes disease: a case-control study

**DOI:** 10.3389/fnut.2024.1501349

**Published:** 2025-01-06

**Authors:** Amr Ali Mohamed Abdelgawwad El-Sehrawy, Bilal AbdulMajeed Mukhlif, Enwa Felix Oghenemaro, M. M. Rekha, Rohit Kumawat, Shilpa Sharma, M. Ravi Kumar, Nagat Salah Shalaby, Munthar Kadhim Abosaoda, Abed J. Kadhim

**Affiliations:** ^1^Department of Internal Medicine, Diabetes Endocrinology and Metabolism, Mansoura University, Mansoura, Egypt; ^2^Medical Laboratory Techniques Department, College of Health and Medical Technology, University of Al-maarif, Anbar, Iraq; ^3^Department of Pharmaceutical Microbiology, Faculty of Pharmacy, Delta State University, Abraka, Delta State, Nigeria; ^4^Department of Chemistry and Biochemistry, School of Sciences, JAIN (Deemed to be University), Bangalore, Karnataka, India; ^5^Department of Neurology, National Institute of Medical Sciences, NIMS University Rajasthan, Jaipur, India; ^6^Chandigarh Pharmacy College, Chandigarh Group of Colleges-Jhanjeri, Mohali, Punjab, India; ^7^Department of Basic Science and Humanities, Raghu Engineering College, Visakhapatnam, India; ^8^College of Nursing, National University of Science and Technology, Nasiriyah, Dhi Qar, Iraq; ^9^College of Pharmacy, The Islamic University, Najaf, Iraq; ^10^Department of Medical Engineering, Al-Nisour University College, Baghdad, Iraq

**Keywords:** diabetes, T2DM, diet, DQI-I, DQI-R

## Abstract

**Background:**

Type 2 diabetes mellitus (T2DM) is a global health crisis linked to increased cardiovascular risk. Research indicates that better dietary quality—higher intake of fruits, vegetables, and whole grains, and lower intake of processed foods—reduces T2DM risk. This study examines the relationship between T2DM and dietary quality indices (DQI-I and DQI-R) to determine if adherence can lower diabetes risk. By analyzing dietary patterns in individuals with and without diabetes, the research aims to identify key nutritional factors influencing disease risk and provide evidence-based dietary recommendations for prevention and management.

**Methods:**

This case-control study involved 128 T2DM patients and 256 controls, assessing dietary intake with a validated 168-item food frequency questionnaire to calculate the Dietary Quality Index-I (DQI-I) and Dietary Quality Index-R (DQI-R). Multivariable logistic regression analysis explored the relationship between DQI-I, DQI-R, and their components with T2DM development odds.

**Results:**

The mean (SD) age and body mass index (BMI) of participants, comprising 53.7% men, were 37.8 (7.8) years and 27.7 (3.3) kg/m^2^, respectively. In the final model, each standard deviation increase in the DQI-I score was associated with reduced odds of T2DM (odds ratio [OR] = 0.61; 95% confidence interval [CI] = 0.37–0.92; *p* = 0.046). Among the components of the DQI-I, a high adequacy score was significantly correlated with lower odds of T2DM (OR = 0.13; 95% CI = 0.05–0.36; *p* < 0.001). Additionally, participants in the highest tertile of the DQI-R score exhibited lower odds of T2DM compared to those in the lowest tertile (OR = 0.29; 95% CI = 0.11–0.49; *p* < 0.001). Furthermore, within the components of the DQI-R, a high moderation score was associated with a decreased risk of T2DM (OR = 0.19; 95% CI = 0.09–0.45; *p* < 0.001).

**Conclusion:**

The case-control study suggests a potential protective effect of diets with higher scores on the Diet Quality Index-International (DQI-I) and Revised Diet Quality Index (DQI-R) in reducing T2DM risk. Future research should focus on larger sample sizes and prospective designs to further investigate the DQI-I, DQI-R, and their components in relation to T2DM and other chronic diseases.

## 1 Introduction

T2DM represents a significant global health challenge characterized by insulin resistance and elevated blood glucose levels, currently impacting 463 million individuals and projected to increase to 700 million by 2045 (1). A comprehensive understanding of its epidemiology and risk factors, including lifestyle and genetic influences, is essential for the development of effective management and prevention strategies (2).

Recent iterations of the Diet Quality Index (DQI), such as the DQI-R, align with contemporary nutritional objectives by evaluating food consumption based on fitness characteristics and their association with chronic disease risks, including type 2 diabetes (3). Elevated DQI scores, which prioritize the consumption of fruits, vegetables, and dietary fibers over processed foods, are linked to a lower risk of diabetes. There is a need for broader coverage and additional research into the dietary impacts on diabetes (4, 5).

The Revised Diet Quality Index (DQI-R) evaluates dietary patterns aimed at reducing the risk of diabetes by integrating contemporary nutritional research and health guidelines, with a focus on plant-based proteins and whole grains (6–8). Higher DQI-R scores correlate with a reduced risk of diabetes, thereby shaping dietary guidelines and informing public health strategies (9, 10).

The examination of atherosclerosis risk in community data indicates that significant weight loss through dietary modifications markedly reduces the risk of cardiovascular disease (CVD) but does not similarly affect the risk of diabetes (11, 12). This underscores the necessity for precise nutritional assessments in the context of CVD prevention (13). Additionally, a study conducted within an Asian population revealed that adherence to healthful dietary patterns, such as the Mediterranean and DASH diets, significantly diminishes the risk of type 2 diabetes, particularly among non-smokers (14).

This study examines the relationship between type 2 diabetes and two DQI (DQI-I and DQI-R) to ascertain whether greater adherence to these indices is linked to a reduced risk of developing diabetes. By analyzing the dietary patterns of individuals with and without diabetes, the research aims to identify critical nutritional components that influence disease risk and to provide evidence-based dietary recommendations for the prevention and management of diabetes. Ultimately, this investigation seeks to enhance global health outcomes through dietary preventive strategies.

Improved infants’ DQI-I and Revised DQI-R scores cannot predict a lower glycemic index level and, as a consequence, a lower propensity to T2DM.

Patients with T2DM who have better DQI-I and DQI-R scores were at lower risk of developing T2DM. It can also involve hypothesized relationships between the scores on the various sub-domains of the DQI-I and DQI-R and T2 DM risk (Such as, higher adequacy score in DQI-I implies a lesser likelihood of T2DM).

## 2 Materials and methods

### 2.1 Study population

This research utilized a case-control study design, concentrating on participants who were between the ages of 18 and 60 years old. These individuals had been diagnosed with type 2 diabetes within the last 6 months, with their diagnosis being based on established criteria related to glucose levels. Specifically, this included fasting blood sugar (FBS) levels of 126 mg/dL or greater and 2-h post-glucose (2 h-PG) levels of 200 mg/dL or greater, as referenced in prior studies (15). In addition to the diabetic participants, the study also incorporated healthy individuals who fell within the same age range, ensuring they met specific glucose level criteria as well. For these healthy participants, their FBS levels were required to be less than 100 mg/dL and their 2 h-PG levels had to be below 200 mg/dL, as also noted in previous research (15). The study established several exclusion criteria to maintain the integrity of the findings. Individuals with certain chronic diseases were not included, nor were those diagnosed with Type 1 diabetes or gestational diabetes. Additionally, participants who were following specific dietary regimens or taking particular medications were excluded from the study. Pregnant women and those who were breastfeeding were also not considered eligible for participation. Moreover, individuals who had a family history of diabetes or hypertension were excluded from the study. Lastly, participants who failed to complete the food frequency questionnaire, which consisted of more than 35 items, or whose reported energy intake was outside the specified range of 800–4,200 kilocalories (16), were also excluded from the analysis.

### 2.2 Dietary assessment

Personal interviews and a semi-quantitative food frequency questionnaire (FFQ) with 168 food items were used to gather data on dietary consumption. Specifically, the year before the case group’s diagnosis and the year before the control group’s interview were the years previously to which the FFQ asked about the frequency of consumption for each item during the previous year. For every food type, participants indicated how often they consumed it (daily, weekly, monthly, or annually). A nutritionist gave instructions on how to use four scales to convert reported food item quantities from home measurements to grams in order to assure measurement uniformity. Every person’s daily food consumption was represented in grams, determined by calculating their intake. A customized version of Nutritionist IV software was used to determine the nutritional makeup of every item.

### 2.3 Calculation of DQI-I

Using the methods developed by Kim et al. (17), the Diet Quality Index-I (DQI-I) was calculated. Higher scores are indicative of a greater food quality; the scoring system goes from 0 to 100. Four main dietary evaluation elements are included in the DQI-I: moderation (0–30 points), overall balance (0–10 points), diversity (0–20 points), and adequacy (0–40 points). The literature (13), for example, provides extensive information on the DQI-I calculation. A score of 0–15 is obtained for variety based on the total diversity of dietary categories, which include dairy, meat, poultry, fish, eggs, beans, grains, fruits, and vegetables. Furthermore, the diversity of protein sources within the category (meat, poultry, fish, dairy, beans, and eggs) adds a score ranging from 0 to 5. The eight components that make up the adequacy score are as follows: fruits, vegetables, grains, fiber, protein, iron, calcium, and vitamin C. Each component has a possible score ranging from 0 to 5. The moderation score was calculated by calculating the scores for total fat, saturated fat, cholesterol, salt, and meals without added calories. Based on predetermined cut-off points, each item was assigned a value between 0 and 6. The fatty acid ratio (Polyunsaturated fatty acids [PUFA]: Monounsaturated fatty acids [MUFA]: Saturated fatty acids [SFA]) and the macronutrient ratio (carbohydrate:protein:fat), which were graded on scales of 0–4 and 0–6, respectively, were used to evaluate the overall balance (13).

### 2.4 Calculation of DQI-R

Haines et al.’s approach was followed in the computation of the DQI-R (18). Throughout their investigation, they included 10 different elements in all, and each element received a score between 0 and 10. These included foods in a variety of forms, with particular attention to total fat, saturated fat, and cholesterol. They also included foods such as fruits, vegetables, grains, calcium, iron, and a varied and moderate diet. The evaluation of four major food categories was used to determine dietary variety, with a maximum score of 2.5 possible for each category. The dietary groupings included of grains, which were further categorized into seven subgroups; vegetables, which were further separated into seven subgroups; fruits, which were categorized into three subgroups; and meat and dairy, which were further divided into seven subgroups. One-quarter of a serving of daily consumption for each individual category was the cut-point used to calculate the score for dietary variety. We also used three particular components to calculate the moderation score, each of which may have a maximum value of 2.5. Added sugars, fats that are optional, and salt consumption were among these elements. The original suggested range of 0 to 10 for the moderation score was changed to a range of 0–7.5 due to the lack of data on alcohol intake pertinent to the idea of “moderation.” As a result, rather than as expected, the overall DQI-R score varied from 0 to 97.5. Previous research has provided extensive information on the DQI-R computation (14).

### 2.5 Statistical analysis

Version 21 of SPSS was used for statistical analysis. Using histograms and the Kolmogorov–Smirnov test, normality was evaluated. For quantitative data, population characteristics were presented as mean ± SD or median (IQR), and for qualitative data, as percentages. Chi-square and t-tests were used to examine the differences between the cases and controls. DQI-I and DQI-R scores were used to divide participants into tertiles. After controlling for covariates, the association between dietary indicators and the likelihood of developing diabetes was investigated using multivariable logistic regression. A significance threshold of *p* < 0.05 was applied to the presented odds ratios and confidence intervals.

## 3 Results

The mean values along with the standard deviation (SD) for the DQI-I and the DQI-R across all populations included in the study were found to be 61.4 ± 5.9. When examining the specific groups, the cases exhibited a mean DQI-I of 61.6 ± 6.3, while the control group had a mean of 61.2 ± 6.1. In terms of the DQI-R, the overall mean was recorded at 73.4 ± 11.9, with cases showing a mean of 73.2 ± 11.4 and controls presenting a mean of 73.6 ± 11.7. The details regarding general demographic information and the scores associated with DQI-I, as well as its individual components measured per 1,000 kilocalories of energy intake, are presented in Table 1 for both cases and controls. Notably, when comparing the diabetes patients to the control group, it was observed that the former had significantly elevated values for Body Mass Index (BMI), percentage of individuals who smoke, family size, instances of foreign travel, income levels, and a more favorable socioeconomic status (SES). Furthermore, the diabetes patients exhibited a lower total variety score, indicating less diversity in their diet, a diminished score reflecting overall food group variety, and a heightened score for the variety of protein sources, with statistical significance indicated by *p* < 0.05.

**Table 1:** Overview of general information and scores of DQI-I and its components for cases and controls.

Variables	Controls (*n* = 256)	Diabetes (*n* = 128)	*p*-value*
Age (years)	38.1 ± 7.9	37.6 ± 7.7	0.325
Male (%)	51.8	55.6	0.258
BMI (Kg/m^2^)	25.3 ± 2.9	30.2 ± 3.7	<0.001
Smoking, (yes, %)	6.2	13.1	0.041
Physical activity (MET/min/week)	1,654 ± 812	1,098 ± 622	<0.001
**Socio economic status (%)**			<0.001
Low (%)	28.7	20.8	
Middle (%)	45.4	45.7	
High (%)	25.9	33.5	
DQI-I components (per 1,000 Kcal)			
***Variety score***	6.41 ± 1.95	6.38 ± 1.75	0.247
Overall food group variety score	5.74 ± 1.60	5.12 ± 1.34	0.002
Variety for protein source score	1.02 ± 0.52	1.25 ± 0.71	0.004
***Adequacy score***	18.3 ± 4.2	16.1 ± 2.9	<0.001
Vegetable group score	2.19 ± 0.89	1.14 ± 0.64	<0.001
Fruit group score	2.65 ± 0.81	1.21 ± 0.61	0.025
Grain group score	2.74 ± 0.51	2.71 ± 0.53	0.091
Fiber score	2.61 ± 0.62	2.09 ± 0.49	0.015
Protein score	2.81 ± 0.78	2.24 ± 0.54	0.048
Iron score	2.74 ± 0.79	2.18 ± 0.48	0.018
Calcium score	2.34 ± 0.62	2.04 ± 0.41	0.027
Vitamin C score	2.27 ± 0.83	1.96 ± 0.54	0.002
***Moderation score***	7.5 ± 3.1	6.1 ± 2.9	0.034
Saturated fat score	0.00 (0.00–1.98)	0.45 (0.00–1.64)	0.547
Cholesterol score	2.41 ± 0.39	2.12 ± 0.41	0.039
Sodium score	0.00 (0.00–1.55)	0.00 (0.00–1.35)	0.148
free-calorie foods score	1.12 (0.00–2.02)	0.00 (0.00–1.54)	0.024
***Overall balance score***	0.41 (0.00–1.45)	0.75 (0.00–1.950)	0.741
Macronutrient ratio (carbohydrate:protein:fat) score	0.00 (0.00–2.15)	0.00 (0.00–2.34)	0.541
Fatty acid ratio (PUFA:MUFA: SFA) score	0.00 (0.00–1.21)	0.00 (0.00–1.15)	0.451
***DQI-I score*** (per 1,000 Kcal)	36.1 ± 7.4	33.2 ± 6.3	0.003

Data are reported mean ± SD or median (25–75 interquartile range) for continuous variables and percentages for categorical variables.

*p-value was determined using the independent two-sample t-test and Chi-square test for continuous and categorical variables, respectively.

The dietary information and scores for the components of the DQI-R and its components (per 1,000 Kcal of energy consumption) for the patients and controls are shown in Table 2. Patients with diabetes had much lower scores for dietary cholesterol, iron, and vegetable intake, along with less variation in their fruit and vegetable intake. Furthermore, these individuals showed reduced discretionary fat and added sugar moderation scores, reduced overall moderation scores, and a poorer DQI-R score overall (*p* < 0.05). In contrast, there was a significant difference (*p* < 0.01) in the energy intake of the case group compared to the control group.

**Table 2:** Overview of general information and scores of DQI-R and its components for cases and controls.

Variables	Controls (*n* = 256)	Diabetes (*n* = 128)	*p*-value*
Dietary intake			
Energy intake (Kcal/d)	2,341 ± 578	2,717 ± 614	0.028
Carbohydrate (% of energy)	54.6 ± 5.8	55.1 ± 6.1	0.855
Protein (% of energy)	13.2 ± 2.2	12.9 ± 2.1	0.247
DQI-R components (per 1,000 Kcal)			
Total fat score	3.12 ± 1.95	3.23 ± 1.75	0.174
Saturated fatty acids score	3.05 ± 1.60	3.12 ± 1.34	0.247
Dietary cholesterol score	3.92 ± 1.17	3.98 ± 1.35	0.455
Fruit intake score	4.17 ± 2.41	4.85 ± 2.67	0.085
Vegetable intake score	3.74 ± 1.89	2.86 ± 1.64	0.039
Grain intake score	2.19 ± 1.66	2.24 ± 1.67	0.417
Calcium intake score	3.20 ± 1.52	3.27 ± 1.57	0.142
Iron intake score	4.31 ± 1.52	4.64 ± 1.71	0.049
***Dietary diversity score***	2.35 ± 0.74	2.64 ± 0.76	0.127
Grains score	0.47 ± 0.11	0.50 ± 0.13	0.351
Vegetable score	0.61 ± 0.13	0.52 ± 0.12	0.025
Fruit score	1.13 ± 0.51	0.95 ± 0.53	0.031
Meat and dairy score	0.73 ± 0.21	0.72 ± 0.21	0.814
***Dietary moderation score***	3.21 ± 1.78	2.24 ± 1.54	0.018
Added sugar score	2.01 ± 0.59	2.32 ± 0.68	0.027
Discretionary fat score	1.24 ± 0.62	0.94 ± 0.41	<0.001
Sodium score	1.31 ± 0.83	1.34 ± 0.54	0.742
***DQI-R score (per 1,000 Kcal)***	39.5 ± 9.1	34.1 ± 8.7	<0.001

Data are presented as mean ± standard deviation.

*p-value was determined using the independent two-sample t-test.

Table 3 shows the association between the chance of diabetes and the DQI-I and its key components. Both the crude and age-and sex-adjusted models showed significant inverse associations between participants in the highest tertiles of DQI-I, variety score, and moderation score and the odds of diabetes; however, these associations did not hold true in the final adjusted model when factors such as body mass index (BMI), smoking status, physical activity, socioeconomic status (SES), and energy intake were taken into account. More specifically, no correlations were seen between the likelihood of having diabetes and greater DQI-I (OR = 0.71, 95% CI [0.35–1.85]; *p*-trend: 0.688), variety score (OR = 0.32, 95% CI [0.11–1.15]; *p*-trend: 0.084), or moderation score (OR = 0.63, 95% CI [0.43–1.79]; *p*-trend: 0.671). The crude model (OR = 0.37, 95% CI: 0.24–0.71; *p*-trend < 0.001), the age and sex-adjusted model (OR = 0.39, 95% CI: 0.21–0.68; *p*-trend < 0.001), and the final adjusted model (OR = 0.26, 95% CI: 0.04–0.57; *p*-trend < 0.001) all showed significantly lower odds of diabetes among participants in the highest tertile of adequacy scores when compared to those in the lowest tertile. However, in all three of the logistic regression models examined, the total balance score did not show a statistically significant correlation with the probabilities of having diabetes.

**Table 3:** Odds ratios (95% confidence intervals) for T2DM across tertiles of the DQI-I and its primary components, as well as per one standard deviation of scores in relation to 1,000 kilocalories of energy intake within the study population.

Variable		Tertiles of DQI-I				
	T1	T2	T3	*p* for trend	Per one SD	*p*-value
DQI-I						
Median score, SD	22.31	29.27	39.78	–	–	–
Crude model	1.00 (Ref)	0.53 (0.39–0.91)	0.51 (0.38–0.90)	0.017	0.72 (0.63–0.95)	0.012
Model 1^*^	1.00 (Ref)	0.53 (0.41–0.92)	0.49 (0.32–0.87)	0.039	0.69 (0.61–0.94)	0.034
Model 2^†^	1.00 (Ref)	0.58 (0.35–1.27)	0.58 (0.24–1.52)	0.134	0.61 (0.37–0.92)	0.046
Variety						
Median score, SD	5.87	7.41	9.45	–	–	–
Crude model	1.00 (Ref)	0.91 (0.63–1.37)	0.61 (0.41–0.96)	0.046	0.84 (0.73–1.07)	0.248
Model 1^*^	1.00 (Ref)	0.89 (0.61–1.34)	0.64 (0.40–0.97)	0.041	0.84 (0.73–1.07)	0.219
Model 2^†^	1.00 (Ref)	0.94 (0.53–1.62)	0.56 (0.33–1.17)	0.081	0.91 (0.62–1.16)	0.572
Adequacy						
Median score, SD	14.27	18.04	22.71	–		–
Crude model	1.00 (Ref)	0.63 (0.39–0.89)	0.41 (0.24–0.63)	<0.001	0.65 (0.57–0.79)	0.028
Model 1^*^	1.00 (Ref)	0.61 (0.37–0.85)	0.39 (0.5–0.61)	<0.001	0.63 (0.73–0.78)	<0.001
Model 2^†^	1.00 (Ref)	0.51 (0.34–0.81)	0.13 (0.05–0.36)	<0.001	0.42 (0.22–0.60)	0.031
Moderation						
Median score, SD	3.04	5.41	10.12	–		–
Crude model	1.00 (Ref)	0.75 (0.52–1.14)	0.71 (0.41–0.97)	0.016	0.78 (0.55–0.93)	0.028
Model 1^*^	1.00 (Ref)	0.79 (0.53–1.25)	0.61 (0.41–0.94)	0.038	0.75 (0.53–0.92)	0.012
Model 2^†^	1.00 (Ref)	1.27 (0.62–1.94)	0.83 (0.46–1.52)	0.344	0.89 (0.59–1.08)	0.379
Overall balance						
Median score, SD	0.00	0.79	1.94	–		–
Crude model	1.00 (Ref)	1.92 (1.24–2.81)	1.23 (0.89–1.82)	0.281	0.93 (0.78–1.11)	0.357
Model 1^*^	1.00 (Ref)	1.96 (1.31–2.93)	1.29 (0.93–1.97)	0.214	0.98 (0.81–1.14)	0.497
Model 2^†^	1.00 (Ref)	2.37 (1.17–3.95)	1.72 (1.05–3.07)	0.119	1.09 (0.92–1.36)	0.254

* Model 1: adjusted for age and sex.

† Model 2: adjusted for model 1 and body mass index, smoking, physical activity, socio-economic status, and dietary intake of energy.

Table 3 shows the odds ratio (OR) for diabetes for each standard deviation (SD) rise in DQI-I and its main components in the context of several adjusted models. In all adjusted models, a one standard deviation increase in DQI-I and adequacy score was linked to a lower probability of having diabetes; in the fully adjusted model, the odds ratio (95% confidence interval) for diabetes per standard deviation increase in DQI-I and adequacy score were 0.61 (0.37–0.92) and 0.42 (0.22–0.60), respectively (*p* < 0.05). A one standard deviation rise in the moderation score was associated with a statistically significant decrease in the likelihood of having diabetes in the crude, age-and sex-adjusted, and final adjusted models (OR = 0.89, 95% CI: 0.59–1.08; *p*-value: 0.379). Nevertheless, this significant connection was no longer present. A one standard deviation increase in variety and overall balance scores did not significantly correlate with the likelihood of having diabetes, according to three logistic regression models. The odds ratios (OR) and 95% confidence intervals (CI) for diabetes per standard deviation increase in overall balance and variety scores in the multivariable model were 1.09 (0.92–1.36) and 0.91 (0.62–1.16), respectively (*p* > 0.05).

Table 4 displays the odds ratios (OR) and 95% confidence intervals (CI) for diabetes across the tertiles of the DQI-R and its constituent parts. Additionally, the scores are shown per standard deviation of scores in connection to an energy intake of 1,000 kcal. In all regression models, lower risks of diabetes were consistently associated with higher DQI-R and dietary moderation scores. In particular, in the crude and final models, the OR (95% CI) for diabetes among individuals in the highest vs. lowest tertiles of DQI-R were 0.42 (0.25–0.61), *p*-trend <0.001, and 0.29 (0.11–0.49), *p*-trend <0.001, respectively. Moreover, in the crude and final adjusted models, the OR (95% CI) for diabetes among individuals in the highest vs. lowest tertiles of the dietary moderation score were 0.39 (0.24–0.66), *p*-trend <0.001, and 0.19 (0.09–0.45), *p*-trend <0.001, respectively. A greater dietary variety score was not linked to an increased risk of developing diabetes in any of the three logistic regression analysis models. The odds ratio (OR) for diabetes in the final adjusted model, comparing the highest to lowest tertile of dietary variety score, was 1.06 (0.49–2.27) with a 95% confidence interval (CI), and the *p*-trend was 0.814.

**Table 4:** Odds ratios (95% confidence intervals) for T2DM across tertiles of the DQI-R and its primary components, as well as per one standard deviation of scores in relation to 1,000 kilocalories of energy intake within the study population.

Variable		Tertiles of DQI-I				
	T1	T2	T3	*p* for trend	Per one SD	*p*-value
DQI-R						
Median score, SD	26.74	37.42	48.17	–	–	–
Crude model	1.00 (Ref)	0.61 (0.38–0.92)	0.42 (0.25–0.61)	<0.001	0.71 (0.53–0.84)	<0.001
Model 1^*^	1.00 (Ref)	0.59 (0.42–0.89)	0.35 (0.16–0.56)	<0.001	0.69 (0.51–0.81)	<0.001
Model 2^†^	1.00 (Ref)	0.46 (0.27–0.82)	0.29 (0.11–0.49)	<0.001	0.52 (0.31–0.72)	<0.001
Dietary diversity						
Median score, SD	2.31	2.98	4.01	–	–	–
Crude model	1.00 (Ref)	0.98 (0.72–1.21)	0.79 (0.51–1.11)	0.369	0.83 (0.70–1.06)	0.126
Model 1^*^	1.00 (Ref)	1.07 (0.79–1.30)	0.83 (0.53–1.18)	0.271	0.81 (0.68–1.02)	0.324
Model 2^†^	1.00 (Ref)	1.22 (0.85–2.50)	1.06 (0.49–2.27)	0.814	0.93 (0.79–1.23)	0.417
Dietary moderation						
Median score, SD	1.96	3.03	4.32	–	–	–
Crude model	1.00 (Ref)	0.47 (0.36–0.71)	0.39 (0.24–0.66)	<0.001	0.58 (0.47–0.70)	<0.001
Model 1^*^	1.00 (Ref)	0.45 (0.33–0.69)	0.37 (0.22–0.62)	<0.001	0.58 (0.47–0.70)	<0.001
Model 2^†^	1.00 (Ref)	0.40 (0.29–0.64)	0.19 (0.09–0.45)	<0.001	0.41 (0.29–0.56)	<0.001

* Model 1: adjusted for age and sex.

† Model 2: adjusted for model 1 and body mass index, smoking, physical activity, socio-economic status, and dietary intake of energy.

Additionally, all adjusted models showed lower chances of diabetes with each standard deviation (SD) rise in the DQI-R and dietary moderation scores (Table 4). The odds ratios (OR) with 95% confidence intervals (CI) for diabetes in the final adjusted model were 0.51 (0.31–0.72), *p* < 0.001 for the DQI-R, and 0.41 (0.29–0.56), *p* < 0.001 for the dietary moderation score. In contrast, none of the logistic models showed a significant correlation between dietary variety and diabetes with a one standard deviation rise in the score. The odds ratio (95% confidence interval) for diabetes per standard deviation increase in dietary variety score was 0.93 (0.77–9.23), *p* = 0.417, in the final adjusted model.

The present case-control study aimed to evaluate the relationship between two indices of dietary quality, namely DQI-I and DQI-R, with T2DM risk. The current study provides evidence to support the hypothesis that better DQI has a protective effect against T2DM. For each standard deviation increase in DQI-I, the odds of T2DM decreased by 39% (OR = 0.61; 95% CI = 0.37–0.92; *p* = 0.046), and this effect was mainly in adequacy (OR = 0.42; 95% CI = 0.22–0.60; *p* < 0.05). In the same manner, subjects with T2DM in the highest tertile of DQI-R had significantly lower odds of T2DM than those in the lowest tertile (OR = 0.29; 95% CI = 0.11–0.49; *p* < 0.001) and the moderation component had the strongest negative association (OR = 0.41; 95% CI = 0.29).

Such outcomes bear testimony to earlier findings that show that diets high in fruits, vegetables, whole grains, and fiber and low in saturated fats, added sugar, and processed foods reduce the risk of diabetes (4, 7, 8, 10). The observed significant association is with the ‘adequacy’ component of DQI-I which emphasizes the need for adequate intake of fruits and vegetables, fiber, proteins, iron, calcium and Vitamin C. The close correlation with the “moderation” factor of DQI-R underlines the necessity of restraining the intake of unhealthy fats, added sugars and sodium.

However, it is important to note the following limitations of this research. Recall bias, especially in terms of diet, is a major drawback of the case control design, although this is an efficient study design for identifying associations. The use of a FFQ, while having been shown to be valid in this study, may have measurement error. The small number of participants may confine the results’ generalization. Moreover, the cross-sectional nature of the data means that it is not possible to establish cause effect relationships; we are only able to determine that higher dietary quality is associated with a lower T2DM risk. Other factors, which could not be controlled for in the multivariate analysis (BMI, smoking, physical activity, SES, energy intake) may still affect the findings.

However, it must be noted that our study has some limitations which must be taken into consideration Since the current study contributes to a growing body of literature on the role of diet quality in T2DM prevention and control, the results of the current study are significant. The consistency of the observed relationships in both the DQI-I and DQI-R, together with strong correlations between the adequacy and moderation components of these indices, supports the call for efforts to improve dietary habits as a key approach to mitigating the impact of T2DM on the global population.

### 3.1 Future research directions

For the purpose of future research, the limitation of this present work should be taken into consideration. Future research should involve larger prospective studies with longer follow-up duration that will provide clearer evidence about causal links between DQI-I, DQI-R and T2DM risk. Better approximations of dietary intake might be realized through the use of more complex dietary assessment tools, for example 24-h dietary recalls or multiple administrations of an FFQ. More research is still required to get more details on the way that dietary quality is associated with T2DM risk, and on the best dietary advice for different population groups and settings. Further research on the co-relationships between dietary quality and other aspects of lifestyle including exercise, genetic makeup will also help to explain more about T2DM causes and how it can be prevented. Last, evaluating the cost-benefit analysis of dietary interventions according to DQI-I and DQI-R would provide direction to the public health policy.

## 4 Discussion

The DQI serves as a critical instrument for investigating the relationship between dietary quality and the risk of type 2 diabetes and other metabolic disorders (19). The DQI provides a comprehensive evaluation of dietary adherence, which is directly linked to health outcomes through the monitoring of food intake and the assessment of compliance with prescribed dietary regimens (20). Elevated DQI scores, indicative of a diet abundant in fruits, vegetables, whole grains, and lean proteins, have consistently been associated with a diminished risk of type 2 diabetes (21). This correlation highlights the potential of a nutrient-dense, well-balanced diet to mitigate the risk of metabolic disorders by facilitating weight management, improving glycemic control, and reducing inflammation.

The significance of the DQI extends beyond diabetes, encompassing a broader spectrum of metabolic disorders, including obesity, hypertension, and dyslipidemia (22). For instance, diets characterized by low DQI scores typically contain elevated levels of salt, saturated fats, and refined carbohydrates, all of which are implicated in the pathophysiology of these conditions (23). Conversely, high DQI scores in dietary assessments indicate adherence to key dietary guidelines, fostering moderation in caloric intake and the consumption of a diverse array of essential nutrients (24). These diets are not only critical for the prevention of type 2 diabetes but also substantially reduce the risk of related metabolic disorders. This underscores the importance of maintaining high dietary quality as an integral component of a comprehensive public health strategy (25). DQI serves as a vital instrument in the ongoing efforts to enhance dietary interventions and foster healthy eating behaviors among individuals, particularly as nutritional patterns evolve globally (26).

The DQI-I and its revised version, the DQI-R, serve as critical tools for evaluating the impact of dietary patterns on the risk of developing type 2 diabetes (27). The DQI-I assesses dietary intake based on four fundamental criteria: variety, adequacy, moderation, and overall balance. It promotes a diverse range of nutrients while restricting the consumption of detrimental components, such as excessive sugars and saturated fats, which are associated with impaired insulin sensitivity and increased obesity risk (28). The DQI-R enhances this approach by placing a stronger emphasis on the quality of fats and carbohydrates rather than merely their quantity, underscoring the detrimental effects of refined sugars and processed foods. By examining these nutritional components, each index delineates dietary patterns that either reduce or elevate the risk of type 2 diabetes, thereby providing a basis for targeted dietary interventions and public health strategies aimed at diabetes prevention and management (29).

Nutritional variety constitutes a fundamental aspect of a balanced diet and overall health, with the DQI-I and DQI-R serving as critical instruments for its assessment and promotion (30). The DQI-I underscores the necessity of consuming a diverse array of nutrients by evaluating dietary intake through the principles of diversity, sufficiency, and balance across multiple food groups. It assesses the inclusion of primary food categories, including fruits, vegetables, grains, protein sources, and dairy, to promote adequate intake of essential vitamins and minerals. To ensure that the diversity in weight-reduction strategies encompasses both high nutritional quality and quantity, the DQI-R enhances this evaluation by prioritizing the healthiest and most beneficial food groups (31). Utilizing both indices facilitates the identification of meals that are both plentiful and adequately nutrient-dense. This approach aids individuals in adopting healthier dietary practices, potentially mitigating nutritional deficiencies and promoting sustained fitness over time (32).

This study, grounded in the DQI-I and DQI-R, investigates the relationship between nutritional diversity and the incidence of type 2 diabetes. The average DQI-I scores for individuals with diabetes and control participants were comparable, suggesting no significant differences in dietary quality. In contrast, the DQI-R scores indicated that control participants exhibited slightly higher dietary quality than those with diabetes, implying that non-diabetic individuals tend to have superior dietary patterns. Among individuals with higher adequacy ratings, a correlation was observed between lower risks of T2DM and reduced dietary diversity, particularly in the consumption of fruits and vegetables. However, the associations between the DQI-I’s variety and moderation scores diminished after adjusting for confounding variables such as BMI and socioeconomic status. Importantly, the dietary diversity score did not demonstrate a significant association with diabetes risk, with odds ratios remaining close to unity. This analysis investigates the relationship between the DQI-I and the DQI-R in the context of T2DM. The mean DQI-I score was similar, with diabetic patients demonstrating a marginally higher score compared to the control group. In contrast, the mean DQI-R score was greater for the control group than for the diabetic cases, suggesting superior dietary quality among non-diabetic individuals. Diabetic patients displayed reduced dietary diversity, particularly in the consumption of fruits and vegetables, alongside elevated Body Mass Index (BMI) and other associated risk factors. Although higher DQI-I scores were initially linked to a decreased likelihood of diabetes, these associations weakened after adjusting for confounding variables, including BMI and socioeconomic status.

The adequacy component of the DQI-I consistently demonstrated a strong association with a reduced risk of diabetes across all adjusted models. In contrast, the overall balance and diversity ratings did not show a substantial correlation with diabetes risk. The consistent association of higher DQI-R scores and dietary moderation with reduced diabetes risk emphasizes the importance of overall dietary quality rather than variety alone in relation to metabolic health. Further research is necessary to comprehensively elucidate these relationships.

Despite the higher SES of diabetic individuals, dietary diversity was significantly lower. This observation contradicts the common expectation that elevated SES correlates with access to a broader array of dietary options. Vos et al. (33), assert that higher SES is typically linked to enhanced dietary choices, as individuals with greater financial resources generally have access to a wider variety of food. The discrepancy in our findings may suggest the influence of behavioral or cultural factors among diabetics that outweigh the financial advantages associated with dietary knowledge.

Consistent with the findings of Yang et al., a significant correlation exists between a diet characterized by low diversity, particularly in fruits and vegetables, and an elevated risk of chronic illnesses, including diabetes (34). The diminished consumption of vegetables, iron, and dietary cholesterol reinforces the hypothesis that individuals with diabetes may require more comprehensive dietary interventions that prioritize essential micronutrients in addition to macronutrients.

The study revealed that despite lower scores on DQI-R notably concerning the moderation of added sugars and fats—individuals with diabetes maintained a high energy intake. This observation implies that the existing dietary guidelines and the framework of the DQI-R may not adequately support optimal nutritional decision-making for this population. Furthermore, Neuhouser et al. highlighted that DQI often do not encompass the comprehensive array of nutritional factors critical for the management of chronic diseases, a limitation that appears to be corroborated by the findings of this study (35).

The adequacy rating of DQI-I emerged as the most significant factor, consistently demonstrating a robust inverse correlation with diabetes across all analytical models. This finding underscores the importance of optimal vitamin intake in mitigating diabetes risk, irrespective of other lifestyle factors. The results of the current study align with the findings of Perraud et al., who posited that sufficient nutrient consumption is essential for preventing recurrent infections. This highlights the necessity for a balanced diet that fulfills all nutritional requirements (36).

When controlling for confounders related to variety and moderation rankings, the lack of significant findings suggests that these factors alone may not serve as adequate indicators of reduced diabetes risk in the presence of other risk factors. This underscores the necessity for a comprehensive integrated approach to dietary assessment and intervention that considers all nutritional supplements and lifestyle factors. The study by Chong and Macpherson corroborates this notion, as they found that physical activity and socioeconomic characteristics often mitigate the influence of dietary intake on health outcomes (37).

Furthermore, the efficacy of dietary adequacy is markedly underscored by the consistent protective effect associated with an increase of one standard deviation in the adequacy score across all models, including the most rigorously adjusted ones. This is true across many different analytical frameworks. Results show that dietary interventions focusing on adequate intake of all essential nutrients rather than on reduction of harmful dietary components are more beneficial. Na and Park (38) emphasize the importance of consuming nutrient-dense meals as opposed to merely restricting caloric intake to mitigate the risk of chronic diseases.

DQI-I and DQI-R are intricately associated with the management and risk of type 2 diabetes through various plausible mechanisms, as indicated by their evaluation of dietary quality. Both indices advocate for a dietary pattern characterized by low saturated fat and high fiber content, which may improve insulin sensitivity and glucose metabolism critical factors in regulating blood sugar levels. The DQI-R, in particular, emphasizes the quality of carbohydrate intake, discouraging excessive consumption of refined sugars and processed grains, which are linked to glycemic fluctuations and an increased risk of diabetes. Both indices promote dietary diversity and sufficiency, ensuring adequate intake of essential micronutrients such as magnesium and chromium, which are critical for glucose metabolism and insulin function. By guiding individuals toward improved dietary patterns, the DQI-I and DQI-R may mitigate infection and oxidative stress, while also reducing the risk of diabetes and facilitating effective disease management.

This research demonstrates several strengths, including a clearly defined sample of individuals aged 18–60, rigorous diagnostic criteria for diabetes, and a comprehensive nutritional assessment employing a detailed food frequency questionnaire. The incorporation of both DQI-I and DQI-R facilitates an in-depth comparison of DQI concerning the risk of T2DM, while accounting for various confounding variables. The case-control methodology, while valuable, inherently limits the ability to establish causal relationships. Additionally, the focus on a narrow age demographic may compromise the generalizability of the findings. The application of exclusion criteria could further restrict the applicability of the results, and reliance on self-reported dietary intake introduces the potential for recall bias. Moreover, the alteration of the moderation score due to incomplete data on alcohol consumption, coupled with the limited range of socioeconomic factors examined, may undermine the robustness of the conclusions drawn. Future investigations should address these limitations through longitudinal studies and broader population sampling to enhance our understanding of the dietary influences on the risk of T2DM.

## 5 Conclusion

In conclusion, the results obtained from this case-control study indicated a potential protective effect associated with a diet characterized by a higher score on DQI-I and the DQI-R in relation to reducing the likelihood of developing TT2DM. It is essential that future research endeavors, which should incorporate larger sample sizes and utilize prospective study designs, focus on examining the DQI-I and DQI-R along with their individual components in connection with T2DM as well as other chronic diseases.

## Data Availability

The raw data supporting the conclusions of this article will be made available by the authors without undue reservation.

## References

[1] Al-Ashoor SGA Ramachandran V Mat LNI Mohamad NA Mohamed MH Sulaiman WAW. Analysis of OCT1, OCT2 and OCT3 gene polymorphisms among type 2 diabetes mellitus subjects in Indian ethnicity. Malaysia Saudi J Biol Sci. (2022) 29:453–9. doi: 10.1016/j.sjbs.2021.09.008, PMID: 35002441 PMC8716931

[2] Ganesh MS. Emerging strategies and effective prevention measures for investigating the association between stroke and sudden cardiac fatality. Curr Cardiol Rev. (2024) 20:35–44. doi: 10.2174/011573403X259676231222053709 38310557 PMC11284691

[3] Kalmpourtzidou A Xinias I Agakidis C Mavroudi A Mouselimis D Tsarouchas A. Diet quality: a neglected parameter in children with food allergies. A cross–sectional study. Front Pediatr. (2021) 9:658778. doi: 10.3389/fped.2021.658778, PMID: 33968858 PMC8102985

[4] Cristello Sarteau A Ercolino G Muthukkumar R Fruik A Mayer-Davis EJ Kahkoska AR. Nutritional status, dietary intake, and nutrition-related interventions among older adults with type 1 diabetes: a systematic review and call for more evidence toward clinical guidelines. Diabetes Care. (2024) 47:1468–1488. doi: 10.2337/dci23-0099 38687466 PMC11362123

[5] Gangwisch JE Hale L St-Onge M-P Choi L LeBlanc ES Malaspina D. High glycemic index and glycemic load diets as risk factors for insomnia: analyses from the women’s health initiative. Am J Clin Nutr. (2020) 111:429–39. doi: 10.1093/ajcn/nqz275, PMID: 31828298 PMC6997082

[6] Diabetes and Nutrition Study Group (DNSG) of the European Association for the Study of Diabetes (EASD). Evidence-based European recommendations for the dietary management of diabetes. Diabetologia. (2023) 66:965–85. doi: 10.1007/s00125-023-05894-8, PMID: 37069434

[7] Chen L-W Fung SM Fok D Leong LP Toh JY Lim HX. The development and evaluation of a diet quality index for Asian toddlers and its perinatal correlates: the GUSTO cohort study. Nutrients. (2019) 11:535. doi: 10.3390/nu11030535, PMID: 30832217 PMC6472180

[8] Dorrington N Fallaize R Hobbs D Weech M Lovegrove JA. Diet quality index for older adults (DQI-65): development and use in predicting adherence to dietary recommendations and health markers in the UK National Diet and nutrition survey. Br J Nutr. (2022) 128:2193–207. doi: 10.1017/S0007114521005043, PMID: 34933704

[9] Koca SB Seber T. The effectiveness of thyroid elastography in evaluating thyroiditis in children with type 1 diabetes. Turkish Arch Pediatr. (2023) 58:322–7. doi: 10.5152/TurkArchPediatr.2023.22323, PMID: 37144267 PMC10210605

[10] de Quadros VP Balcerzak A Allemand P de Sousa RF Bevere T Arsenault J. Global trends in the availability of dietary data in low and middle-income countries. Nutrients. (2022) 14:2987. doi: 10.3390/nu14142987, PMID: 35889943 PMC9324857

[11] Lu L Jing W Qian W Fan L Cheng J. Association between dietary patterns and cardiovascular diseases: a review. Curr Probl Cardiol. (2024) 49:102412. doi: 10.1016/j.cpcardiol.2024.102412 38278463

[12] Crupi AN Haase J Brandhorst S Longo VD. Periodic and intermittent fasting in diabetes and cardiovascular disease. Curr Diab Rep. (2020) 20:1–14. doi: 10.1007/s11892-020-01362-4, PMID: 33301104

[13] Xu Z Steffen LM Selvin E Rebholz CM. Diet quality, change in diet quality and risk of incident CVD and diabetes. Public Health Nutr. (2020) 23:329–38. doi: 10.1017/S136898001900212X, PMID: 31511110 PMC6992481

[14] Chen Guo Chong CG Koh Woon Puay KW Nithya Neelakantan NN Yuan Jian Min YJ Qin LiQiang QV Dam RV. Diet quality indices and risk of type 2 diabetes mellitus. Am. J. Epidemiol. (2018). 10.1093/aje/kwy183 PMC6887680 30165478

[15] Wang J Thornton JC Bari S Williamson B Gallagher D Heymsfield SB. Comparisons of waist circumferences measured at 4 sites. Am J Clin Nutr. (2003) 77:379–84. doi: 10.1093/ajcn/77.2.379, PMID: 12540397

[16] Committee IR. Guidelines for data processing and analysis of the international physical activity questionnaire (IPAQ)-short and long forms. (2005). Available at: http://www.ipaq.ki.se/scoring.pdf

[17] Kim S Haines PS Siega-Riz AM Popkin BM. The diet quality index-international (DQI-I) provides an effective tool for cross-national comparison of diet quality as illustrated by China and the United States. J Nutr. (2003) 133:3476–84. doi: 10.1093/jn/133.11.3476, PMID: 14608061

[18] Haines PS Siega-Riz AM Popkin BM. The diet quality index revised: a measurement instrument for populations. J Am Diet Assoc. (1999) 99:697–704. doi: 10.1016/S0002-8223(99)00168-6 10361532

[19] Abaj F Sotoudeh G Karimi E Rafiee M Koohdani F. Interaction between the dietary indices and PPAR-γ pro 12Ala gene variants on cardiovascular risk factors in patients with type 2 diabetes mellitus. Int J Clin Pract. (2021) 75:e14307. doi: 10.1111/ijcp.14307, PMID: 33930247

[20] De Miguel-Etayo P Moreno LA Santabárbara J Martín-Matillas M Azcona-San Julian MC Del Moral AM. Diet quality index as a predictor of treatment efficacy in overweight and obese adolescents: the EVASYON study. Clin Nutr. (2019) 38:782–90. doi: 10.1016/j.clnu.2018.02.032, PMID: 29730135

[21] Daneshzad E Larijani B Azadbakht L. Diet quality indices and cardiovascular diseases risk factors among diabetic women. J Sci Food Agric. (2019) 99:5926–33. doi: 10.1002/jsfa.9867, PMID: 31206677

[22] Tao W Wang G Wei J. The role of chitosan oligosaccharide in metabolic syndrome: a review of possible mechanisms. Mar Drugs. (2021) 19:501. doi: 10.3390/md19090501, PMID: 34564163 PMC8465579

[23] Yki-Järvinen H Luukkonen PK Hodson L Moore JB. Dietary carbohydrates and fats in nonalcoholic fatty liver disease. Nat Rev Gastroenterol Hepatol. (2021) 18:770–86. doi: 10.1038/s41575-021-00472-y, PMID: 34257427

[24] Martinchik AN. Indices of diet quality as a tool for integrated assessment of dietary intake. Vopr Pitan. (2019) 88:5–12. doi: 10.24411/0042-8833-2019-10024, PMID: 31265770

[25] Puri S Shaheen M Grover B. Nutrition and cognitive health: a life course approach. Front Public Health. (2023) 11:1023907. doi: 10.3389/fpubh.2023.1023907, PMID: 37050953 PMC10083484

[26] Hlaing-Hlaing H Pezdirc K Tavener M James EL Hure A. Diet quality indices used in Australian and New Zealand adults: a systematic review and critical appraisal. Nutrients. (2020) 12:3777. doi: 10.3390/nu12123777, PMID: 33317123 PMC7763901

[27] Esmaeily Z Sotoudeh G Rafiee M Koohdani F. Apo A2–256T> C polymorphism interacts with healthy eating index, dietary quality index-international and dietary phytochemical index to affect biochemical markers among type 2 diabetic patients. Br J Nutr. (2022) 127:1343–51. doi: 10.1017/S0007114521002348, PMID: 34167597

[28] Segovia-Siapco G Paalani M Oda K Pribis P Sabaté J. Associations between avocado consumption and diet quality, dietary intake, measures of obesity and body composition in adolescents: the teen food and development study. Nutrients. (2021) 13:4489. doi: 10.3390/nu13124489, PMID: 34960040 PMC8708967

[29] Zhao Q Wu Q Zhong H Yan B Wu J Guo W. Association of dietary habits with body mass index and waist circumference, and their interaction effect on hypertension. Medicine. (2024) 103:e38178. doi: 10.1097/MD.0000000000038178, PMID: 38758876 PMC11098196

[30] Zheng J Zhu T Li F Wu H Jiang S Shivappa N. Diet quality and mortality among Chinese adults: findings from the China health and nutrition survey. Nutrients. (2023) 16:94. doi: 10.3390/nu16010094, PMID: 38201925 PMC10780502

[31] Pfisterer KJ Amelard R Keller HH Wong A. Characterizing Canadian long-term care home consumed foods and their inflammatory potential: a secondary analysis. BMC Public Health. (2023) 23:261. doi: 10.1186/s12889-022-14934-8, PMID: 36747181 PMC9903425

[32] Wright EC Van Oort B Bjøntegaard MM Carlsen MH Andersen LF. Environmental and nutritional assessment of young children’s diets in Norway: comparing the current diet with national dietary guidelines and the EAT-lancet reference diet. Eur J Nutr. (2023) 62:3383–96. doi: 10.1007/s00394-023-03243-4, PMID: 37653070 PMC10611869

[33] Vos M Deforche B Van Kerckhove A Michels N Geuens M Van Lippevelde W. Intervention strategies to promote healthy and sustainable food choices among parents with lower and higher socioeconomic status. BMC Public Health. (2022) 22:2378. doi: 10.1186/s12889-022-14817-y, PMID: 36536355 PMC9761028

[34] Yang W Yang Y He L Zhang M Sun S Wang F. Dietary factors and risk for asthma: a Mendelian randomization analysis. Front Immunol. (2023) 14:1126457. doi: 10.3389/fimmu.2023.1126457, PMID: 36911739 PMC9992976

[35] Neuhouser ML. The importance of healthy dietary patterns in chronic disease prevention. Nutr Res. (2019) 70:3–6. doi: 10.1016/j.nutres.2018.06.002, PMID: 30077352 PMC6328339

[36] Perraud E Wang J Salomé M Huneau J-F Lapidus N Mariotti F. Plant and animal protein intakes largely explain the nutritional quality and health value of diets higher in plants: a path analysis in French adults. Front Nutr. (2022) 9:924526. doi: 10.3389/fnut.2022.924526, PMID: 35836593 PMC9274246

[37] Chong TW Macpherson H. Pounding the pavement: is the path to brain health steeper for people experiencing greater socioeconomic deprivation? J Alzheimers Dis. (2024) 99:117–20. doi: 10.3233/JAD-240095 38640159

[38] Na K Park YJ. Protein restriction in metabolic health: lessons from rodent models. Nutrients. (2024) 16:229. doi: 10.3390/nu16020229, PMID: 38257122 PMC10819042

